# 3-hydroxyanthranilic acid suppresses colorectal cancer growth and enhances 5-fluorouracil sensitivity via the YY1-p53 and YY1-DKK4 axes

**DOI:** 10.3389/fphar.2026.1928654

**Published:** 2026-09-21

**Authors:** Guifang Gan, Chengfang Shangguan, Yuan Yuan, Suoyu Xiang, Zhaopeng Shi

**Affiliations:** 1 Department of Clinical Laboratory Medicine, Shanghai Ninth People’s Hospital, Shanghai Jiao Tong University School of Medicine, Shanghai, China; 2 Department of Oncology, Rui Jin Hospital, Shanghai Jiao Tong University School of Medicine, Shanghai, China; 3 Department of Pathology, Shanghai Ninth People’s Hospital, Shanghai Jiao Tong University School of Medicine, Shanghai, China; 4 Basic Medical Institute, Key Laboratory of Cell Differentiation and Apoptosis of the Chinese Ministry of Education, Shanghai Jiao Tong University School of Medicine, Shanghai, China; 5 Department of Histoembryology, Genetics and Developmental Biology, Key Laboratory of Cell Differentiation and Apoptosis of the Chinese Ministry of Education, Shanghai Jiao Tong University School of Medicine, Shanghai, China

**Keywords:** 3-hydroxyanthranilic acid, 5-fluorouracil, chemoresisitance, colorectal cancer, Dickkopf Wnt signaling pathway inhibitor 4

## Abstract

**Introduction:**

The tryptophan metabolite 3-hydroxyanthranilic acid (3-HAA) has been reported to exert tumor-growth-inhibitory activity; however, its functional role and underlying mechanisms in colorectal cancer (CRC) remain largely unexplored.

**Methods:**

We measured 3-HAA levels in CRC tissues and evaluated its effects on CRC cell proliferation through exogenous administration. Mechanistic investigations were performed to identify its direct protein targets and downstream signaling pathways. The therapeutic potential of 3-HAA, alone and in combination with 5-fluorouracil (5-FU), was assessed both *in vitro* and *in vivo* using cell line-derived xenograft mouse model and AOM/DSS-induced colorectal tumor mouse model.

**Results:**

3-HAA exhibited depleted levels in CRC tissues, and its exogenous administration effectively suppressed the proliferation of multiple CRC cell lines. Through its binding to the Yin Yang 1 (YY1) transcription factor, it disrupted the YY1-p53 interaction, preserved p53 stability, and induced cell cycle arrest. Notably, *in vitro*, 3-HAA reduced chemoresistance to the common CRC-chemotherapy drug 5-fluorouracil (5-FU) via the YY1-DKK4 axis. Consistently, *in vivo*, combined 3-HAA-5-FU treatment effectively inhibited CRC progression.

**Discussion:**

Our findings demonstrate that 3-HAA functions as a tumor suppressor in CRC by targeting the YY1–p53 axis and enhancing chemosensitivity to 5-FU. These results highlight the potential of 3-HAA as a promising therapeutic agent for CRC treatment.

## Introduction

Despite screening-, surgical-, and therapy-related advances, colorectal cancer (CRC), a prevalent digestive-system malignancy, remains majorly responsible for cancer-associated morbidity and mortality (Wu et al., 2025; Sung et al., 2021), with 5-year survival rates for advanced or metastatic CRC often below 15% (Favier et al., 2025; Dekker et al., 2019). Central to systemic CRC therapy (Heidelberger et al., 1957), the “thymineless death” -inducer 5-fluorouracil (5-FU) inhibits *de novo* thymidine synthesis (van der Wilt and Peters, 1994). However, 30%–50% of CRC patients have developed intrinsic or acquired resistance (Hammond et al., 2016), severely hampering its efficacy and highlighting the significance of more effective CRC-targeted clinical strategies.

The kynurenine (KYN) pathway, which catabolizes ≈95% of dietary tryptophan (TRP) (Badawy, 2017), produces diverse bioactive metabolites through its indoleamine 2,3-dioxygenase 1 (IDO1) and tryptophan 2,3-dioxygenase (TDO2) initiator enzymes (Cervenka et al., 2017), with 3-hydroxyanthranilic acid (3-HAA) as a central metabolite owing to its immunomodulatory, redox-active, and signaling properties (Darlington et al., 2010; Krause et al., 2011). With limited understanding of the role of 3-HAA in CRC, despite reports on its suppression of hepatocellular carcinoma growth and sensitizing effects towards targeted therapies such as sorafenib (Gan et al., 2021), we demonstrated that 3-HAA exhibited depleted levels in CRC tissues and that its exogenous administration effectively inhibited tumor progression. Through its binding to the YY1 transcription factor, 3-HAA disrupted the YY1-p53 interaction, thus inhibiting proliferation through p53 stabilization and cell-cycle-arrest (CCA) induction. Furthermore, *in vitro* and *in vivo,* 3-HAA, downregulating Dickkopf Wnt signaling pathway inhibitor 4 (*DKK4*) via YY1, reduced the 5-FU resistance of CRC, making it a promising CRC therapy.

## Materials and methods

### Experimental animals

Originated from Shanghai Model Organisms Center, Inc. (China) and provided *ad libitum* food and water access, 6-week-old male BALB/c nude mice (18–22 g) and C57BL/6 mice were housed under specific pathogen-free conditions and a 12-h light/dark cycle. All mice were humanely euthanized. First, mice were deeply anesthetized with 5% isoflurane (R510-22, RWD, Shenzhen, China) in the induction chamber, and then death was confirmed by cervical dislocation.

### Cell lines and culture conditions

Sourced from ATCC and the Cell Bank of the Chinese Academy of Sciences, HCT116, HT29, and SW620 cells were cultured in supplemented DMEM (L110KJ, Basal Media, Shanghai, China) containing 10% fetal bovine serum (FSP500, ExCell Bio, Suzhou, China) and penicillin-streptomycin solution (S110JV, Basal Media, Shanghai, China). These cultures, regularly inspected for *mycoplasma* contamination, were maintained at 37 °C under 5% CO_2_ enriched humidified conditions.

### Cell proliferation and viability assessment

Following seeding (1 × 10^5^ cells) in 6-well plates [day 0 (D0)], on days 4 and 7 (D4 and D7), cells were stained with 0.4% trypan blue (40207ES20, Yeasen, Shanghai, China) and counted using a hemocytometer, with three independent experiments for each sample.

### Immunohistochemical (IHC) analysis

Prepared via tissue fixation in 4% paraformaldehyde (G1101, Servicebio, Wuhan, China) and embedding in paraffin (39601006, Leica, Wetzlar, Germany), 5 µm tissue sections were subjected to deparaffinization; rehydration; and 20-min, 95 °C antigen retrieval in citrate buffer (P0083, Beyotime, Shanghai, China). Then, they had their endogenous peroxidase blocked with 3% H_2_O_2_ (KIT-9710, MXB, Fuzhou, China) and were incubated overnight at 4 °C with anti-3-HAA (IS001, ImmuSmol, Bordeaux, France), anti-DKK4 (A7797, Abclonal, Wuhan, China) and anti-cleaved caspase-3 (A11021, Abclonal, Wuhan, China) primary antibodies, followed by 1-h, room-temperature incubation with a 1:500 HRP-conjugated secondary antibody (KIT-9710, MXB, Fuzhou, China). Following diaminobenzidine (DAB)-based, signal-development treatment, the sections were counterstained with hematoxylin (G1140, Solarbio, Beijing, China).

### RNA sequencing (RNA-seq) and differential expression analysis

Established with the TruSeq Stranded mRNA Library Prep Kit (20020594, Illumina, San Diego, United States), libraries derived from extracted total RNA [Total RNA Isolation Kit (RC112-01), Vazyme, Nanjing, China] were sequenced (paired-end 150 bp) on an Illumina NovaSeq 6,000 platform. Genes showing |log_2_ fold change| > 1 and adjusted *p* < 0.05 in DESeq2-based differential expression analysis were considered differentially expressed genes (DEGs). Data is stored in the GEO repository (GSE338052, 338053).

### Quantitative real-time PCR (qPCR)

Following complementary DNA (cDNA) synthesis using the ABScript Neo RT Master Mix (RK20433, Abclonal, Wuhan, China), mixing with Universal SYBR Green Fast qPCR Mix (RK21203, Abclonal, Wuhan, China), and analysis on a CFX Opus 96 Real-Time PCR System (12011319, Bio-Rad, Hercules, United States), relative gene expression levels, quantified with the 2^−ΔΔCt^ method, were normalized to GAPDH. The primer sequences were as follows: *CDC6*-F: AGA​AGG​GCC​CCA​TGA​TTG​TG, *CDC6*-R: TCC​TGC​AAA​CAT​CCA​GTG​CT; *CDC45*-F: GGG​CCA​TCG​TTG​GAC​TAA​CA, *CDC45*-R: GGG​AAG​ACC​CAT​GTC​TGC​AA; *CDK2*-F: TGC​ATC​TTT​GCT​GAG​ATG​GTG, *CDK2*-R: TGG​CTG​GCC​AAG​ACT​AGA​AG; *CDK4*-F: CCT​GTG​TCT​ATG​GTC​GGG​C, *CDK4*-R: CAG​TCG​CCT​CAG​TAA​AGC​CA; *GADD45A*-F: CAC​TGT​CGG​GGT​GTA​CGA​AG, *GADD45A*-R: GTT​GAT​GTC​GTT​CTC​GCA​GC; *TP53*-F: TGA​CAC​GCT​TCC​CTG​GAT​TG, *TP53*-R: GCA​GGG​GAG​TAC​GTG​CAA; *FTH1*-F: TCA​ACA​GTG​CTT​GGA​CGG​AA, *FTH1*-R: GTC​CTG​GTG​GTA​GTT​CTG​GC; *NQO1*-F: GAG​TCC​CTG​CCA​TTC​TGA​AA, *NQO1*-R: ATG​TCC​CCG​TGG​ATC​CCT​T; *KITLG*-F: GTC​CCC​GGG​ATG​GAT​GTT​TT, *KITLG*-R: GGC​CTT​CCC​TTT​CTC​AGG​AC; *DKK4*-F: GCC​GAT​TTC​ACG​CAC​CTT​AC, *DKK4*-R: ACC​CAC​GAC​ATG​TAG​CAC​AG; and *RGS5*-F: GTG​CAA​AGG​ACT​TGC​AGC​TT, *RGS5*-R: TTC​TCA​GCC​ATC​TTG​GCA​GG.

### Cell cycle analysis

After trypsinization-based harvesting and two ice-cold PBS washes, cells fixed in 70% ethanol (overnight, 4 °C treatment) were stained (30 min, 37 °C, in the dark) with 50 μg/mL propidium iodide staining solution (P4864, Sigma-Aldrich, St. Louis, United States) and analyzed for cell cycle distribution [CytoFLEX Flow Cytometer (Beckman Coulter, United States) and FlowJo software].

### Western blot analysis

After cell or tissue lysis in RIPA buffer (PC101, Epizyme, Shanghai, China) containing protease inhibitor cocktails (MB2678-2, MeilunBio, Dalian, China) and subsequent protein quantification with the BCA assay (ZJ101, Epizyme, Shanghai, China), proteins (40 µg) separated by SDS-PAGE were transferred onto PVDF membranes (IPVH00010, Millipore, Boston, United States), followed by membrane blocking and overnight, 4 °C membrane incubation with primary antibodies against pCDC2 [4,539, Cell Signaling Technology (CST), Danvers, United States], CDC2 (9,116, CST, Danvers, United States), GADD45A (4,632, CST, Danvers, United States), p53 (A19585, Abclonal, Wuhan, China), YY1 (A19569, Abclonal, Wuhan, China), DKK4 (A7797, Abclonal, Wuhan, China), KITLG (A5672, Abclonal, Wuhan, China), HA (AE008, Abclonal, Wuhan, China), phospho-c-JUN (AP0105, Abclonal, Wuhan, China), c-JUN (A11378, Abclonal, Wuhan, China), cleaved caspase-3 (A11021, Abclonal, Wuhan, China), and GAPDH (AC033, Abclonal, Wuhan, China). Afterward, these membranes were treated with 1:10,000 HRP-conjugated secondary antibodies (LF102, Epizyme, Shanghai, China) and then with an enhanced chemiluminescence (ECL) substrate (MA0186-2, MeilunBio, Dalian, China) for signal development.

### Co-immunoprecipitation (Co-IP) assays

After cell lysis in IP buffer (PC105, Epizyme, Shanghai, China) containing protease inhibitors (MB2678-2, MeilunBio, Dalian, China) and subsequent overnight, 4 °C incubation of the resulting lysates with Anti-HA Magnetic Beads (88836, Thermo Fisher Scientific, Waltham, United States) or IgG magnetic beads (AC044N, Abclonal, Wuhan, China), the immunoprecipitated complexes were eluted through two consecutive steps—bead washing with the IP buffer (four times) and bead boiling in 2× SDS loading buffer (LT103, Epizyme, Shanghai, China)—and were then analyzed by Western blot.

### Plasmid construction and transfection

Using Lipofectamine 3000 (L3000015, Invitrogen, Carlsbad, United States), 70%–80% confluence cells were transfected with the pLenti-YY1, pLenti-DKK4, pLenti-KITLG, and pGIPZ-YY1-KD1/2 plasmids, as well as the packaging vectors psPAX2 and pMD2.G; 6 hours after which, the transfection medium was replaced with fresh complete medium.

### Dual-luciferase reporter assays

Forty-eight hours after Lipofectamine 3000-assisted cell co-transfection with 100 ng of a firefly luciferase reporter plasmid containing either wild-type or mutant putative binding sites, 10 ng of a Renilla luciferase control plasmid (pRL-TK, Promega, United States), and 100 ng of the pSG5-YY1 plasmid or an empty vector, luciferase activity measurement (E1910, Dual-Luciferase Reporter Assay System, Promega, Madison, United States; with firefly luciferase activity normalized to the Renilla counterpart) was performed on lysates of transfected cells in Passive Lysis Buffer.

### Chromatin immunoprecipitation (ChIP)-PCR assays

Following protein-DNA cross-linking via cell treatment with 1% formaldehyde (F8775, Sigma-Aldrich, St. Louis, United States, 10 min, room temperature) and subsequent reaction quenching via 0.125 M glycine (G01623, ABCONE, Shanghai, China) addition, the resulting chromatin was ultrasonically sheared (B01080010, Bioruptor Pico, Diagenode, Belgium) into 200–600 bp fragments. Through two consecutive incubation steps—overnight, 4 °C incubation with 2 µg of anti-YY1 antibody (61779, Active Motif, Carlsbad, United States) or normal rabbit IgG (A7016, Beyotime, Shanghai, China, as a negative control), followed by 2-h incubation with Pierce Protein A/G Magnetic Beads (88802, Thermo Fisher Scientific, Waltham, United States), protein-DNA complexes were captured. After sequential washing, elution, and cross-link reversal, DNA was purified for PCR-based semi-quantification of enrichment using primers flanking the putative binding sites (ChIP-F: TCC​TGA​CCT​CAG​GTG​ATC​CA; ChIP-R: AAC​TCC​AAT​GGC​CGG​GCA​CA).

### Cell line-derived xenograft mouse model construction

After subcutaneous injection of HCT116 cells (2 × 10^6^ cells in 100 µL of PBS), into the flanks of BALB/c nude mice, tumors were measured with a digital caliper every 3 days. When they reached 80–120 mm^3^ [tumor volume = (length × width^2^)/2], the mice (*n* = 6 per group) were intraperitoneally injected with 3-HAA (148776, Sigma-Aldrich, St. Louis, United States, 100 mg/kg per day) and/or 5-FU (T0984, TargetMol, Boston, United States, 20 mg/kg every 3 days) for 2 weeks. At the experimental endpoint, tumors were excised from euthanized mice, weighed, and used for subsequent analyses.

### AOM/DSS-induced colorectal tumour mouse model

To induce colitis-associated CRC, mice received a single intraperitoneal injection of azoxymethane (AOM, MB5521-1, MeilunBio, Dalian, China) at a dose of 10 mg/kg body weight. One week later, colitis was initiated by administering 2% (w/v) dextran sulfate sodium (DSS, 60316ES76, Yeasen, Shanghai, China) in the drinking water for 7 consecutive days, followed by 14 days of regular water for recovery. This cycle was repeated 3 times in total. For therapeutic intervention, mice (*n* = 6 per group) were intraperitoneally injected with 3-HAA (100 mg/kg per day) and/or 5-FU (20 mg/kg every 3 days) for 2 weeks. At the experimental endpoint, mice were euthanized, and the entire colon was excised, opened longitudinally. Colonic length was measured, and macroscopic tumors were counted. The colon tissues were then fixed in 4% paraformaldehyde (G1101, Servicebio, Wuhan, China), embedded in paraffin (39601006, Leica, Wetzlar, Germany), and sectioned at 5 µm thickness. Following deparaffinization and rehydration, the sections were stained with haematoxylin and eosin (318906, Sigma-Aldrich, St. Louis, United States) for histopathological examination.

### Statistical analysis

Between-group differences in results from ≥3 independent experiments [mean ± standard deviation (SD)] were evaluated using an unpaired, two-tailed Student’s *t*-test in GraphPad Prism, with *p* < 0.05 considered statistically significant.

## Results

### 3-HAA suppressed CRC cell growth (CRC-CG)

Highly active in tumor tissues, the KYN pathway (Figure 1A) is responsible for metabolizing ≈95% of TRP in the human body (Platten et al., 2019). To investigate CRC-related TRP metabolic alterations, we first examined the expression levels of key enzymes in this pathway using the TCGA database and assessed the clinical relevance of their mRNA levels. In CRC tissue vs. normal tissue comparisons, IDO1 and TDO2, which catalyze TRP-to-KYN conversion, were upregulated, whereas kynurenine 3-monooxygenase (KMO) and hydroxyanthranilate 3,4-dioxygenase (HAAO)were downregulated (Figures 1A,B). This coordinated expression pattern indicates KYN pathway activation and reduced 3-HAA production, prompting direct measurements of 3-HAA levels in CRC tissues. Through IHC analysis, 3-HAA levels were confirmed to be lower in CRC tissues than in adjacent non-cancerous counterparts (Figure 1E). Notably, significant viability reductions in HCT116, HT29, and SW620 cells were observed upon exogenous administration of 50 μM and 100 μM 3-HAA, whereas other TRP metabolites—KYN, picolinic acid (PA), and quinolinic acid (QA)—did not produce comparable effects (Figures 1C,D). These findings indicate the unique CRC-CG inhibitory activity of 3-HAA with respect to the tested TRP metabolites.

**FIGURE 1:**
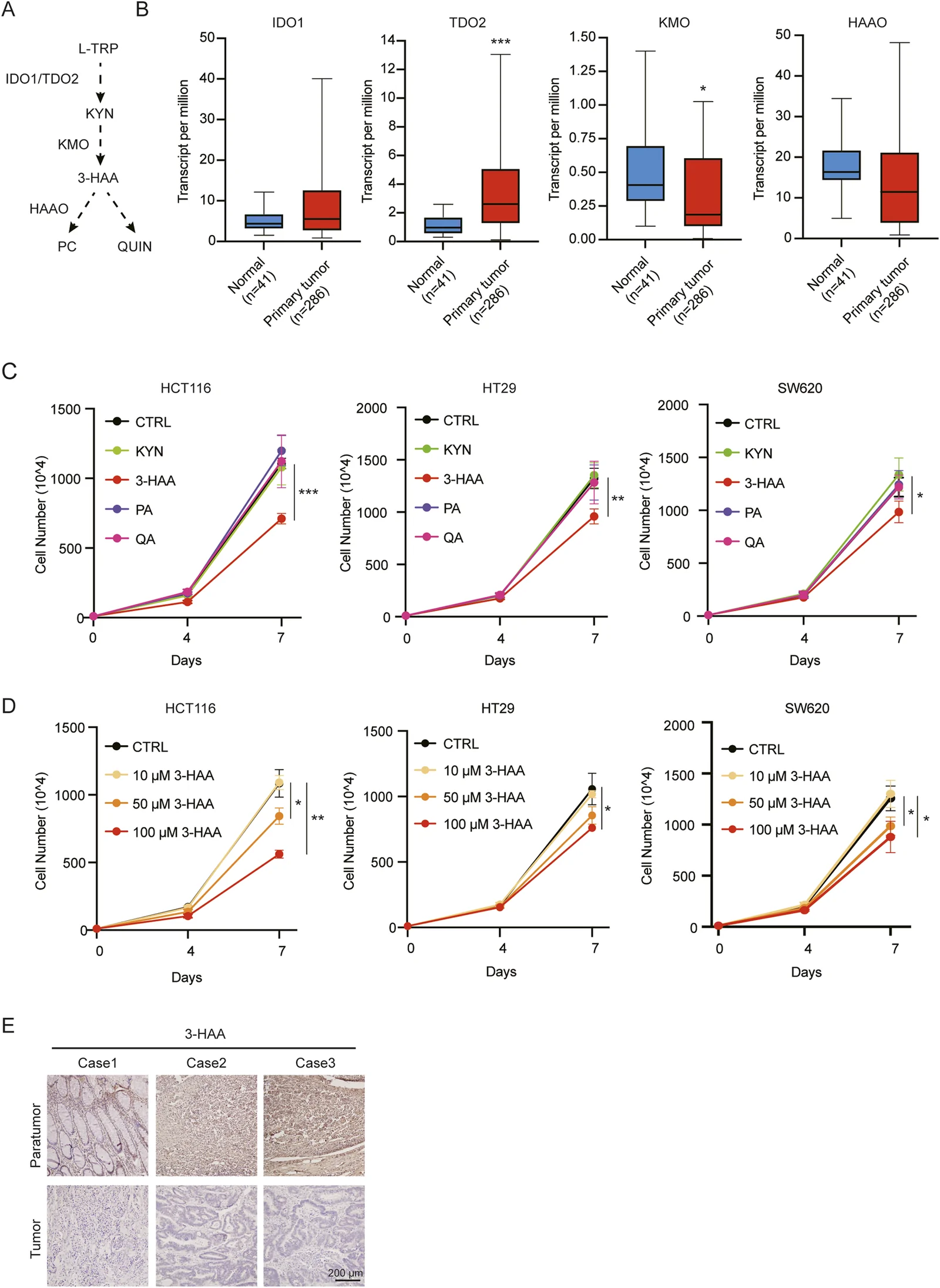
3-HAA is reduced in CRC tissues and suppresses CRC-CG. **(A)** A schematic diagram of the TRP metabolic pathway. **(B)** Differential expression of TRP metabolic enzymes between CRC tissues and adjacent normal tissues. Data were obtained from the TCGA database (portal.gdc.cancer.gov), and results are presented as the mean ± SD. *: *p* < 0.05, ***: *p* < 0.001. **(C)** 3-HAA, but not other TRP metabolites, inhibits CRC-CG. CRC cells were treated with 100 μM KYN, 3-HAA, PA, or QA for 7 days. Results are presented as the mean ± SD (*n* = 3), with *p*-values determined by a two-tailed *t*-test. *: *p* < 0.05, **: *p* < 0.01. **(D)** Dose-dependent inhibition of CRC-CG by 3-HAA. Results are presented as the mean ± SD (*n* = 3), with *p*-values determined by a two-tailed *t*-test. *: *p* < 0.05, **: *p* < 0.01. **(E)** Representative IHC staining of 3-HAA in CRC and adjacent non-cancerous tissues.

### 3-HAA induced G2/M CCA in CRC cells

To elucidate the mechanism underlying 3-HAA mediated CRC-CG inhibition, we performed RNA-seq based transcriptional profiling of 3-HAA treated HCT116 cells. Only minimal gene-expression changes were observed after 24-h treatment (Supplementary Figure S1A), with most DEGs associated with the aryl hydrocarbon receptor (AHR) pathway (Supplementary Figure S1B). While this finding suggested the mediating role of AHR signaling in the 3-HAA inhibitory effect, neither agonism nor antagonism of this pathway affected cell growth (Supplementary Figure S1C), indicating that AHR signaling is not the primary mediator of the observed growth inhibition.

Given the lack of evident transcriptional changes after 24-h treatment, we extended the treatment duration to 7 days and observed a substantial increase in DEGs (Figures 2A,B). Both KEGG pathway analysis and gene set enrichment analysis (GSEA) indicated associations between these transcriptional changes and cell cycle regulation (Figures 2C–E). This observation was supported by qPCR findings, which showed that 3-HAA significantly upregulated *GADD45A* expression while downregulating key cell-cycle regulators, including *CDC45*, *CDC6*, *CDK2*, and *CDK4*, without altering *TP53* mRNA levels (Figure 2F).

**FIGURE 2:**
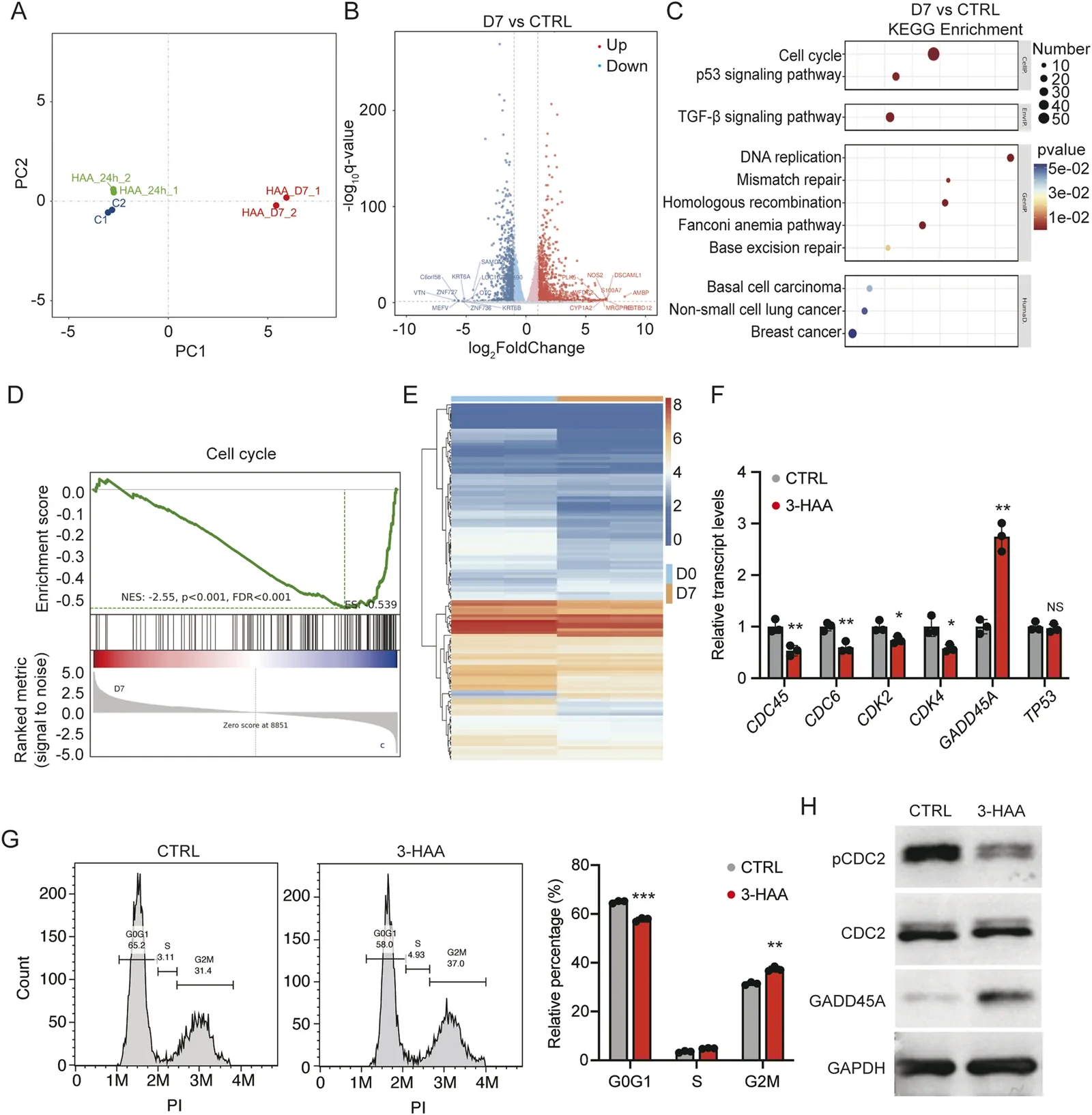
3-HAA induces G2/M CCA in CRC cells. **(A)** Principal component analysis (PCA) of HCT116 cells treated with 100 μM 3-HAA for 24 h or 7 days. **(B)** 3-HAA alters global gene expression profiles in HCT116 cells treated with 100 μM 3-HAA for 7 days. **(C)** KEGG pathway analysis of upregulated and downregulated genes. **(D–F)** 3-HAA modulates the expression of cell-cycle-related genes. **(D)** GSEA of cell-cycle-associated genes, **(E)** heatmaps of differentially expressed cell-cycle-related genes, and **(F)** qPCR validation. HCT116 cells were treated with 100 μM 3-HAA for 7 days. Results are presented as the mean ± SD (*n* = 3), with *p*-values determined by a two-tailed *t*-test. *: *p* < 0.05, **: *p* < 0.01. **(G,H)** 3-HAA induces G2/M phase arrest in CRC cells. HCT116 cells were treated with 100 μM 3-HAA. **(G)** Representative histograms showing cell cycle distribution and **(H)** Western blot analysis of GADD45A, p-CDC2, and total CDC2. Results are presented as the mean ± SD (*n* = 3), with *p*-values determined by a two-tailed t-test. **: *p* < 0.01, ***: *p* < 0.001.

Cell-cycle-distribution analysis using PI staining showed that 3-HAA treatment elevated the proportion of G2/M CRC cells (Figure 2G) while reducing the G0/G1 population. Supporting these findings, Western blot analysis demonstrated reduced CDC2 phosphorylation (Tyr15) and increased GADD45A expression in HCT116 cells (Figure 2H). Overall, these findings indicate that 3-HAA suppresses CRC cell proliferation by inducing G2/M CCA.

### YY1 mediated 3-HAA induced p53 stabilization and CCA

We observed that 3-HAA induced changes in p53 signaling pathway (Figure 2C), and many differentially expressed cell cycle related genes were found to be associated with p53 (Figure 2F). Our analysis of the 3-HAA effect on p53 showed that the TRP metabolite promoted p53 accumulation (Figure 3A). Based on our previous finding that 3-HAA is a YY1 agonist ligand (Shi et al., 2021), together with reports that YY1 facilitates p53 degradation (Furlong et al., 1996; Gronroos et al., 2004; Sui et al., 2004), we hypothesized that 3-HAA stabilizes p53 by disrupting the YY1-p53 interaction.

**FIGURE 3:**
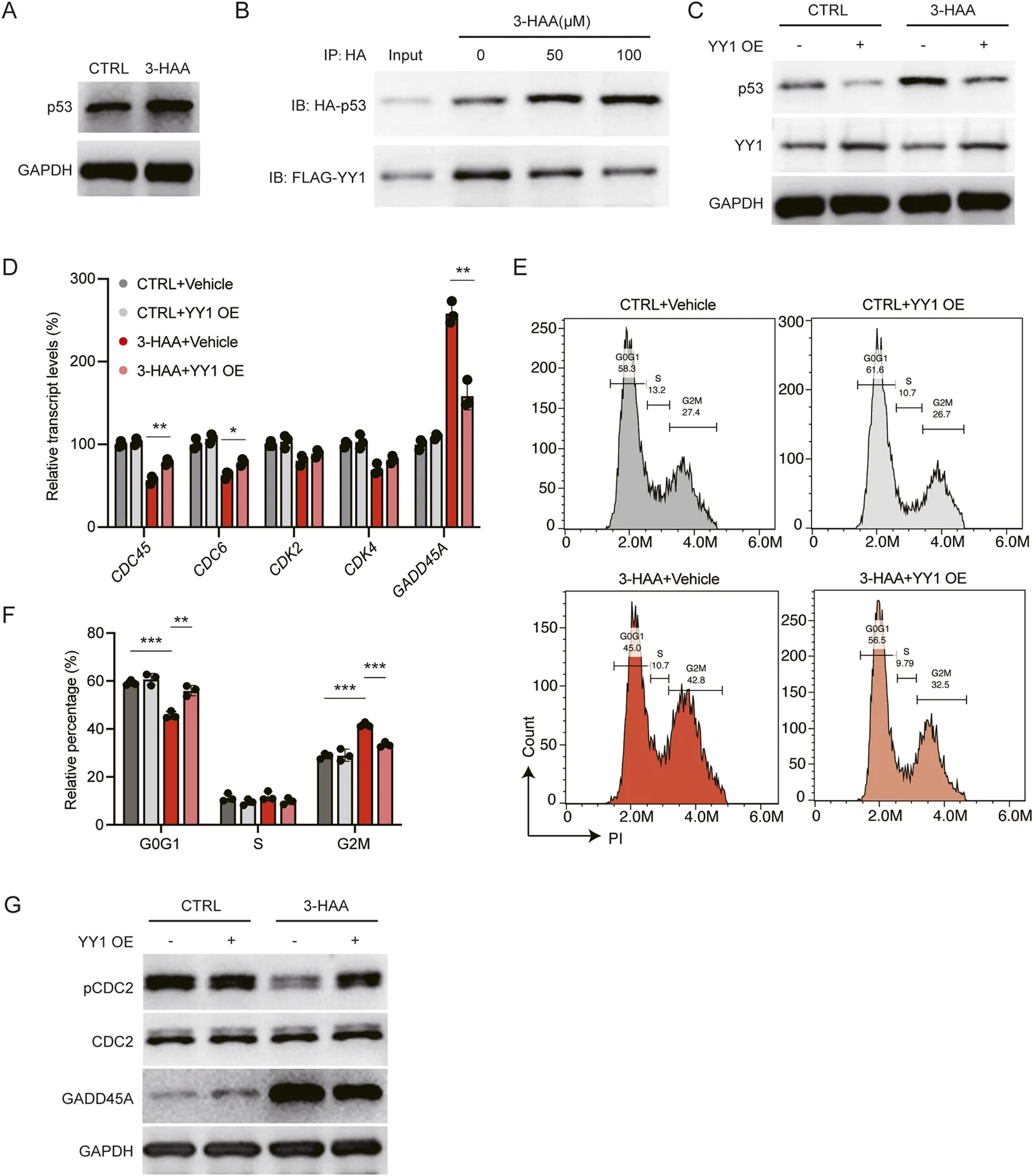
3-HAA induces CCA by disrupting the YY1-p53 interaction in CRC cells. **(A)** 3-HAA promotes p53 accumulation. HCT116 cells were treated with 100 μM 3-HAA for 7 days. **(B)** 3-HAA reduces the YY1-p53 interaction. Co-IP analysis was performed to assess the interaction between p53 and YY1 in cells treated with or without 3-HAA for 4 days. **(C)** 3-HAA prevented p53 level reductions induced by YY1 overexpression. p53 expression was examined by Western blot. **(D)** YY1 reverses 3-HAA induced changes in cell-cycle-related gene expression. Cells were treated with or without 100 μM 3-HAA for 7 days. QPCR results are presented as the mean ± SD (*n* = 3), with *p*-values determined by a two-tailed *t*-test. *: *p* < 0.05, **: *p* < 0.01. **(E–G)** YY1 rescues 3-HAA induced G2/M CCA. **(E)** Representative histograms and **(F)** quantitative analysis of cell cycle distribution. Results are presented as the mean ± SD (*n* = 3), with *p*-values determined by a two-tailed *t*-test. **: *p* < 0.01, ***: *p* < 0.001. **(G)** Western blot analysis of GADD45A, p-CDC2, and total CDC2.

Supporting this hypothesis, Co-IP analysis demonstrated a reduced YY1-p53 interaction following 3-HAA treatment (Figure 3B). Moreover, the treatment prevented p53 level reductions induced by YY1 overexpression (Figure 3C), indicating that 3-HAA stabilizes p53 by modulating its interaction with YY1.

Investigation into the YY1 role in 3-HAA induced cell cycle regulation demonstrated that YY1 overexpression reversed the 3-HAA mediated upregulation of *GADD45A* and downregulation of *CDC45* and *CDC6* (Figure 3D), counteracting the 3-HAA effect on G2/M phase arrest in HCT116 cells (Figures 3E,F). Moreover, the YY1 overexpression restored phosphorylated CDC2 (p-CDC2) levels and attenuated 3-HAA induced GADD45A accumulation (Figure 3G). Collectively, these findings demonstrate that 3-HAA induces G2/M CCA by targeting YY1.

### 3-HAA enhanced 5-FU sensitivity in CRC by downregulating *DKK4* expression

Considering the role of 5-FU as a cornerstone chemotherapeutic agent for CRC, we next investigated whether 3-HAA could enhance its therapeutic efficacy. Notably, combined 3-HAA-5-FU treatment produced significantly greater CRC-CG suppression compared with either agent alone (Figure 4A).

**FIGURE 4:**
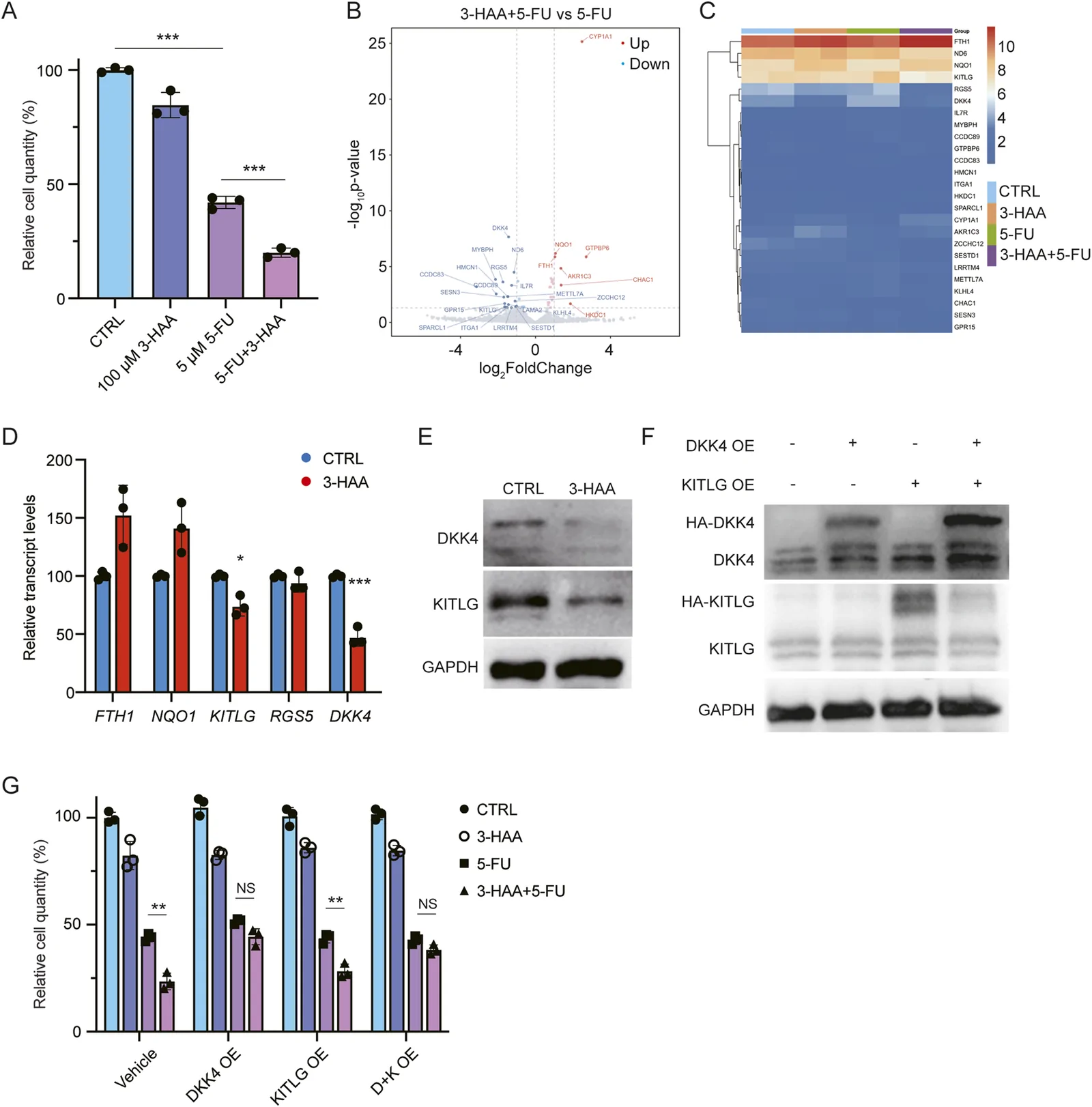
3-HAA enhances 5-FU sensitivity in CRC cells by downregulating *DKK4* expression. **(A)** 3-HAA enhances CRC cell sensitivity to 5-FU. HCT116 cells were treated with 100 μM 3-HAA and/or 5 μM 5-FU for 4 days. Results are presented as the mean ± SD (*n* = 3), with *p*-values determined by a two-tailed *t*-test. ***: *p* < 0.001. **(B–D)** 3-HAA alters the gene expression profile of CRC cells under 5-FU treatment. **(B)** Volcano plots showing DEGs between the 3-HAA + 5-FU and 5-FU alone groups, **(C)** heatmaps of DEGs across treatment groups, and **(D)** qPCR validation of selected genes. HCT116 cells were treated with 100 μM 3-HAA and/or 5 μM 5-FU for 4 days. Results are presented as the mean ± SD (*n* = 3), with *p*-values determined by a two-tailed t-test. *: *p* < 0.05, ***: *p* < 0.001. **(E)** 3-HAA downregulates DKK4 and KITLG expression. HCT116 cells were treated with or without 100 μM 3-HAA for 4 days. **(F)** Ectopic overexpression of DKK4 and KITLG in HCT116 cells. **(G)** DKK4 overexpression reverses the sensitizing effect of 3-HAA on 5-FU treatment. HCT116 cells were treated with 100 μM 3-HAA and/or 5 μM 5-FU for 4 days. Results are presented as the mean ± SD (*n* = 3), with *p*-values determined by a two-tailed *t*-test. **: *p* < 0.01.

A mechanistic RNA-seq based analysis of 3-HAA and 5-FU treated CRC cells identified *FTH1*, *NQO1*, *KITLG*, *RGS5*, and *DKK4* as the most prominently altered genes, as visualized by volcano plots and heatmaps (Figures 4B,C). Subsequent qPCR validation revealed that only *KITLG* and *DKK4* were significantly downregulated following 3-HAA treatment (Figure 4D), a result confirmed at the protein level by Western blot analysis (Figure 4E). These findings determined KITLG and DKK4 as candidate mediators in the 5-FU sensitizing effect of 3-HAA.

To further characterize the functional relevance of the two significantly downregulated genes, we generated CRC cells overexpressing either *KITLG* or *DKK4* (Figure 4F). In contrast to the negligible effect of *KITLG* overexpression, *DKK4* overexpression abolished the 5-FU sensitizing ability of 3-HAA (Figure 4G), indicating that 3-HAA enhances the 5-FU sensitivity of CRC cells through *DKK4* downregulation.

### 3-HAA repressed *DKK4* transcription through YY1 dependent regulation

Given the dual functions of YY1 as a transcriptional activator and repressor, we hypothesized that 3-HAA modulates its transcriptional activity at the *DKK4* promoter. Using JASPAR (jaspar.genereg.net), we identified two potential YY1 binding sites within the *DKK4* promoter (at positions −799 to −794 and −598 to −593; Figure 5A) and evaluated their functional relevance using luciferase reporter assays. To determine whether YY1 regulates *DKK4* expression through these sequences, the corresponding promoter fragments were cloned into luciferase reporter constructs, and YY1-dependent luciferase activity was measured. Notably, constructs containing the full-length *DKK4* promoter exhibited reduced luciferase activity, whereas mutation at the two predicted YY1 binding sites (mut1/2) significantly increased luciferase activity (Figure 5A), indicating that YY1 directly binds to these regions to repress *DKK4* transcription.

**FIGURE 5:**
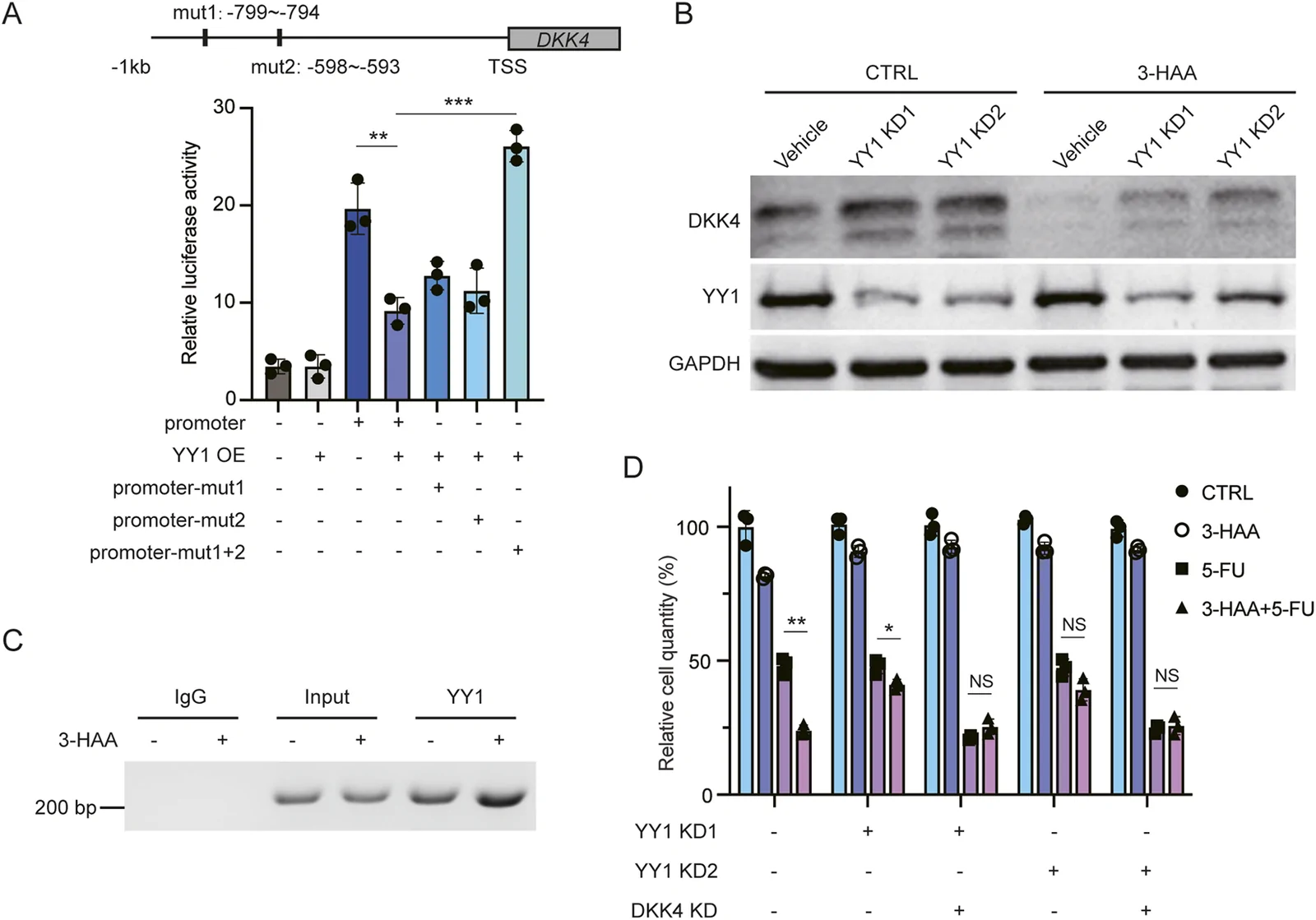
3-HAA represses *DKK4* transcription through YY1-dependent promoter binding. **(A)** YY1 represses *DKK4* promoter activity. A luciferase reporter assay was performed. Results are presented as the mean ± SD (*n* = 3), with *p*-values determined by a two-tailed *t*-test. **: *p* < 0.01, ***: *p* < 0.001. **(B)** YY1 knockdown (KD) reverses 3-HAA induced downregulation of DKK4. YY1 KD HCT116 cells were treated with or without 100 μM 3-HAA for 4 days. **(C)** YY1 directly binds to the *DKK4* promoter. HCT116 cells were treated with or without 100 μM 3-HAA for 4 days, and ChIP-PCR analysis was performed. **(D)** 3-HAA reduces CRC cell resistance to 5-FU via the YY1-DKK4 axis. Cells were treated with 100 μM 3-HAA and/or 5 μM 5-FU for 4 days. Results are presented as the mean ± SD (*n* = 3), with *p*-values determined by a two-tailed *t*-test. *: *p* < 0.05, **: *p* < 0.01.

Consistently, YY1 knockdown reversed the 3-HAA inhibitory effect on *DKK4* expression (Figure 5B), while ChIP-PCR results confirmed the direct YY1-*DKK4* promoter binding (Figure 5C). Moreover, YY1 depletion reduced 3-HAA’s ability to increase the 5-FU sensitivity of CRC cells, with this reduction primarily mediated by *DKK4* (Figure 5D), thereby identifying the YY1-DKK4 axis as a critical pathway through which 3-HAA modulates chemoresistance in CRC.

Aiming to elucidate how *DKK4* downregulation enhances 5-FU sensitivity, we hypothesized that *DKK4* regulates 5-FU resistance via JNK pathway modulation, as DKK4 is known to suppress JNK signaling and caspase-3 activation (Lou et al., 2021). Consistent with this hypothesis, *DKK4* knockdown increased JUN phosphorylation (Fig. S2A). Conversely, a JNK inhibitor reversed the growth inhibition, JNK signaling activation, and caspase-3 cleavage observed in 5-FU treated *DKK4* knockdown cells (Supplementary Figures S2B,C). These results suggest that DKK4 confers 5-FU resistance by suppressing JNK signaling.

### 3-HAA suppressed tumor growth and potentiated 5-FU efficacy *in vivo*

Evaluating the *in vivo* therapeutic potential of 3-HAA, we established HCT116 xenografts in nude mice. When tumors reached 80–120 mm^3^, mice were administered 3-HAA (100 mg/kg per day) and/or 5-FU (20 mg/kg every 3 days) for 2 weeks, with tumor growth monitored every 3 days. At the experimental endpoint, tumors excised from euthanized mice were weighed.

Consistent with *in vitro* results, despite significant tumor-volume-and-weight reductions upon 3-HAA treatment, combined 3-HAA-5-FU therapy produced a more pronounced inhibitory effect compared to either agent alone (Figures 6A–D). Histological analysis further supported these observations, as IHC staining revealed reduced DKK4 expression following 3-HAA treatment and increased cleaved caspase-3 levels in the combined therapy group (Figure 6E). To further validate these findings, we utilized an AOM/DSS induced colorectal cancer model (Figure 6F). In this model, combined administration of 3-HAA and 5-FU effectively alleviated intestinal damage induced by AOM/DSS (Figures 6G–J). H&E staining demonstrated a reduction in tumor burden across treatment groups. The control group displayed dense, contiguous tumor parenchyma with severe mucosal damage and glandular atrophy. Both monotherapy groups showed partial tumor regression with residual focal lesions. In contrast, the combination therapy nearly eliminated tumor foci, with the tissue architecture resembling that of normal colon (Figure 6H). Collectively, these *in vivo* findings highlight the 3-HAA’s potential as a therapeutic agent for CRC.

**FIGURE 6:**
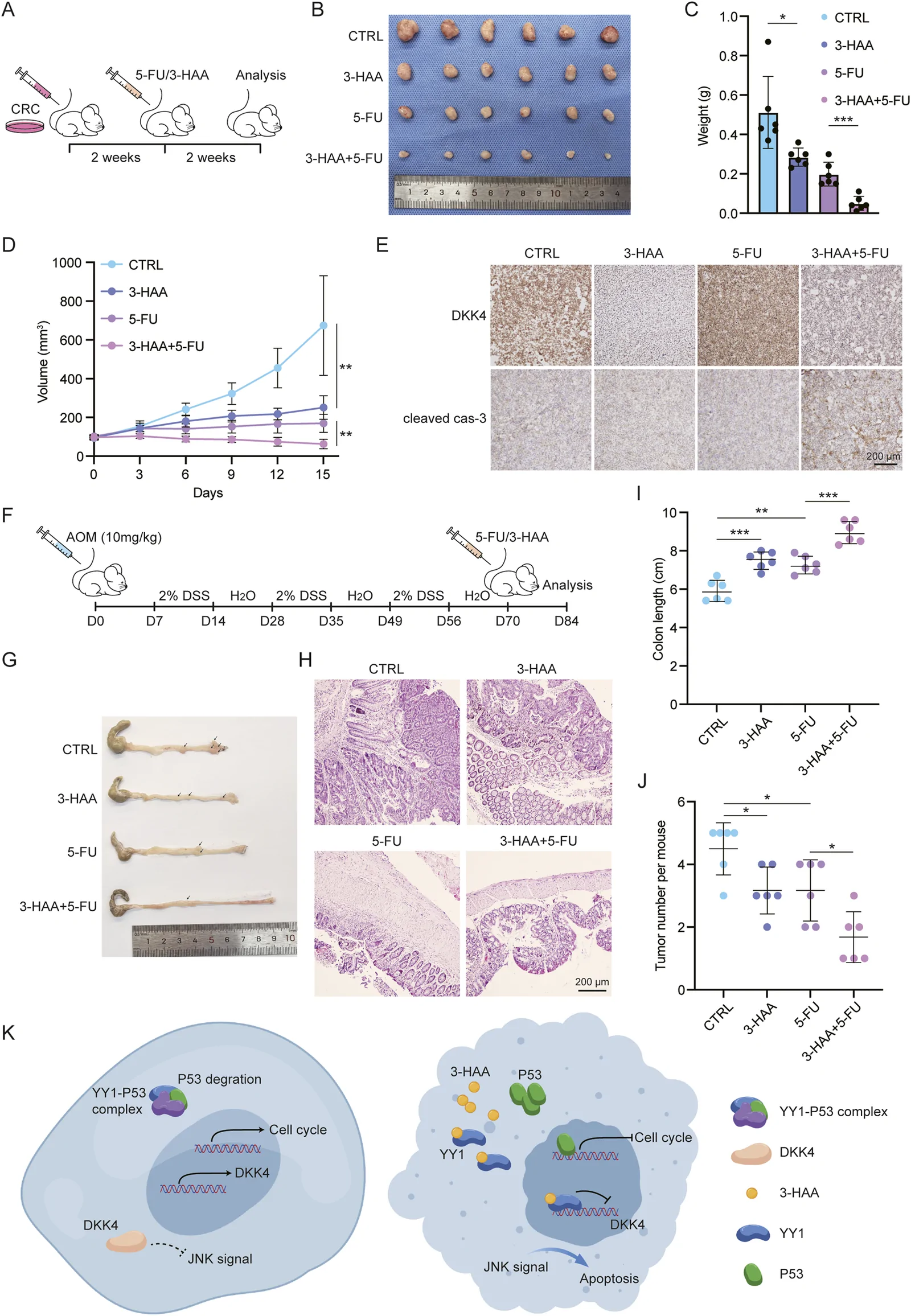
3-HAA suppresses tumor growth and enhances 5-FU efficacy *in vivo*. **(A)** Experimental design for establishing and treating HCT116 xenograft tumors in nude mice. **(B)** Representative images of excised tumors from each group. **(C)** Endpoint tumor weights presented as the mean ± SD (*n* = 6). *: *p* < 0.05, ***: *p* < 0.001. **(D)** Tumor growth curves showing tumor volume over time following treatment initiation. Data are presented as the mean ± SD (*n* = 6). **: *p* < 0.01. **(E)** IHC analysis of DKK4 and cleaved caspase-3 expression in tumor sections. **(F)** Experimental design for establishing intestinal tumors in C57 mice. **(G)** Representative luminal views of the colons from different drugs treated mice. The arrows point to the tumors. **(H)** H&E staining of colon sections. **(I)** Colon length of each group. Data are presented as the mean ± SD (*n* = 6). **: *p* < 0.01, ***: *p* < 0.001. **(J)** Number of colorectal tumors. Data are presented as the mean ± SD (*n* = 6). *: *p* < 0.05. **(K)** The working model of 3-HAA enhancing 5-FU sensitivity in CRC.

## Discussion

Our finding that 3-HAA suppressed CRC growth and reduced chemoresistance prompted a re-evaluation of its upstream metabolic regulation within the KYN pathway. In this pathway, which catabolizes ≈95% of dietary TRP, IDO and TDO function as rate-limiting enzymes (Wang et al., 2026), while the metabolite KYN facilitates tumor immune evasion (Fong et al., 2023). KYN is further metabolized by KMO into several products, including 3-HAA (Shi et al., 2022). Given that 3-HAA inhibited tumor growth (Figure 1C), we hypothesized that KMO exerts dual antitumor effects: it reduces KYN levels, thereby increasing tumor susceptibility to immune surveillance, and elevates 3-HAA production, thus directly suppressing tumor proliferation. These dual functions suggest the diagnostic or prognostic value of KMO expression in CRC, warranting further investigation.

Compared with other tryptophan-derived metabolites involved in colorectal cancer progression, 3-HAA exhibits distinct biological activities and functional outputs. Kynurenine, the major upstream product of the kynurenine pathway, acts predominantly as a pro-tumorigenic mediator in CRC: it activates aryl hydrocarbon receptor (AHR) signaling to drive tumor-cell proliferation, stemness maintenance, and Treg-dominated immunosuppression within the tumor microenvironment (Liu et al., 2025; Yang et al., 2024). Kynurenic acid exerts context-dependent effects; at low concentrations it may confer anti-inflammatory protection for intestinal epithelium, whereas elevated levels support CRC cell survival and counteract oxidative stress-driven cell death, showing limited direct tumor-suppressive potency against established CRC lesions. By contrast, indole-3-lactic acid generally confers anti-tumor properties by remodeling CD8^+^ T-cell-dependent anti-tumor immunity, yet it rarely triggers direct cytotoxic in CRC cells (Lu et al., 2024). In contrast to these metabolites, 3-HAA exhibits a distinctive dual capacity: it directly suppresses the growth of CRC cells, while concurrently modulating the immune microenvironment within the tumor. This unique activity is rarely observed among other tryptophan catabolites.

In recent years, multiple emerging pre-clinical therapeutic candidates have been developed for CRC treatment, including small-molecule metabolic inhibitors, metabolic-targeted interventions, and novel immunotherapeutic agents. Small-molecule glycolysis or glutamine-metabolism inhibitors impair tumor nutrient utilization and partially restore anti-tumor T-cell function; however, their efficacy is often offset by metabolic compensation and off-target toxicity in normal intestinal tissues (Zhu et al., 2025). Metabolic-modulating strategies targeting the kynurenine-AHR axis aim to relieve AHR-mediated immune suppression, but single-agent anti-tumor activity remains modest in most microsatellite stable (MSS) CRC pre-clinical models (Guangzhao et al., 2025). Next-generation immune candidates, including immune-checkpoint agonists and T-cell-engaging bispecific antibodies, achieve prominent responses in mismatch-repair-deficient CRC, while therapeutic benefits are largely restricted in the MSS CRC subtype (Andac-Aktas and Calibasi-Kocal, 2025). Unlike these candidates, 3-HAA offers an additional advantage through its synergistic interaction with 5-FU, significantly amplifying the drug’s therapeutic efficacy in MSS CRC (Miyashita et al., 2024).

Governing multiple cell-cycle-progression stages, the critical tumor suppressor p53 induces CCA by inhibiting CDK4/6 and CDC6 (Kudo et al., 2024; Hall et al., 2007) while directly activating *GADD45A* (Hou et al., 2025). YY1 negatively regulates p53 through dual mechanisms: reducing its transcriptional activity and promoting its degradation (Furlong et al., 1996; Gronroos et al., 2004; Sui et al., 2004), thereby improving tumor cell survival. Building on this framework, our results demonstrated that 3-HAA, acting as a YY1 ligand (Shi et al., 2021), disrupted the YY1-p53 interaction, leading to p53 stabilization and subsequent CCA induction (Figure 3). In addition, 3-HAA downregulated cell-cycle-related genes *CDC45* and *CDC6* (Figure 2F), whose promoters contain YY1 binding sites (JASPAR), suggesting that 3-HAA coordinates gene expression through a combined YY1- and p53-dependent regulatory mechanism.

More than 6 decades after its introduction, 5-FU still constitutes a core drug in CRC chemotherapy; it acts as a cytotoxic antimetabolite by restraining thymidylate synthase activity and entering DNA and RNA as nucleotide-analogue species (Singh et al., 2025). However, drug resistance, toxicity, and tumor heterogeneity frequently limit its clinical efficacy, necessitating more effective combination strategies. In this context, we demonstrated both *in vitro* and *in vivo* that 3-HAA significantly enhanced 5-FU antitumor efficacy, with this effect mediated through CCA induction and *DKK4* downregulation. These findings support the viability of incorporating 3-HAA into standard 5-FU based regimens to overcome chemoresistance in CRC patients.

Extending previous findings by Ebert et al. (Ebert et al., 2012) that transcription factor AP-2 epsilon (TFAP2E) is a key DKK4 upstream regulator and that TFAP2E epigenetic silencing promotes DKK4 derepression mediated chemoresistance, our results identified an additional regulatory layer: 3-HAA induced *DKK4* downregulation via YY1-dependent transcriptional repression. Classically recognized as a Wnt/β-catenin pathway inhibitor, DKK4 is increasingly implicated in non-canonical Wnt signaling pathways, including the JNK pathway (Lou et al., 2021; Hirata et al., 2011). Consistently, our findings demonstrated that *DKK4* knockdown restored JNK signaling and the 5-FU sensitivity of CRC cells (Supplementary Figure S2). Nevertheless, the precise JNK-signaling regulatory mechanisms of DKK4—whether through interactions with specific receptors (e.g., ROR2, RYK, or Frizzled receptors) or via alternative intermediates—require further clarification.

Consistent with the lack of a detectable inhibitory effect on CRC-CG during the first 4 days of treatment (Figure 1), 3-HAA did not produce significant transcriptional changes after 24 h (Supplementary Figure S1). In contrast, 7-day treatment resulted in substantial gene expression changes. This delayed cellular response suggests that 3-HAA requires active cellular transport; however, the specific transporter(s) responsible for its uptake have not been identified. The mechanism underlying 3-HAA uptake into CRC cells therefore warrants further investigation.

Pharmacokinetic data for 3-HAA in humans are currently lacking, with its metabolic fate primarily derived from animal studies suggesting rapid metabolism and excretion. Toxicological data are also limited, with most evidence coming from animal models indicating a relatively low toxicity profile at certain doses (Gan et al., 2021; Dang et al., 2023). However, the safety of long-term use and its clinical therapeutic window remain unknown. This is a critical gap for future clinical translation. Therefore, rigorous Phase I clinical trials are critically warranted to precisely establish these pharmacokinetic parameters.

Despite the promising results, several limitations warrant consideration. First, our study was conducted in mouse, which may not fully recapitulate the complexity of the human tumor microenvironment. Our findings require further validation in patient-derived organoid (PDO) models and larger clinical cohorts. Second, the sample size was relatively small, potentially limiting the statistical power to detect rare adverse events or subtle subgroup differences. Finally, comprehensive toxicity assessments were not performed in this study, and the long-term safety profile of the intervention requires further validation. Our ongoing efforts are focused on establishing PDO biobanks and conducting comprehensive biomarker analyses to bridge the gap between bench and bedside. In addition, future research should focus on translating these findings into clinical settings and exploring other synergistic therapeutic combinations. A particularly promising avenue is the integration of 3-HAA with immunotherapy. Studies have shown that 3-HAA can regulate the immune cell and cytokine secretion network in the tumor microenvironment (Xue et al., 2023). While immunotherapy has an unsatisfactory therapeutic effect on most patients with CRC (Han et al., 2024; Yousefi et al., 2026), we will evaluate the efficacy of combining our agent with anti-PD-1/PD-L1 or anti-CTLA-4 antibodies to enhance the effect of immunotherapy.

In conclusion, our findings demonstrated that 3-HAA stabilized p53 by disrupting the YY1-p53 interaction, thereby inducing CCA in CRC cells, and concurrently reduced resistance to 5-FU through the YY1-DKK4 axis (Figure 6K). Collectively, these results position 3-HAA as a promising therapeutic candidate for CRC clinical management.

## Data Availability

The data presented in the study are deposited in the GEO repository, accession number GSE338052, GSE338053.

## Supplementary material

SUPPLEMENTARY FIGURE S1 3-HAA does not inhibit CRC-CG through the AHR signaling pathway. **(A)** 3-HAA induces minimal gene expression changes at early time points. HCT116 cells were treated with or without 100 μM 3-HAA for 24 hours. **(B)** 3-HAA modestly upregulates a subset of AHR target genes. HCT116 cells were treated with or without 100 μM 3-HAA for 24 hours. Results are presented as the mean + SD (n = 3), with *p*-values determined by a two-tailed t-test. *: *p* < 0.05. **(C)** Modulation of AHR signaling does not affect CRC-CG. HCT116 cells were treated with or without CH-223191 (AHR inhibitor, AHRi) and L-kynurenine (AHR agonist, AHRa).

SUPPLEMENTARY FIGURE S2 DKK4 knockdown enhances 5-FU sensitivity by activating JNK signaling in CRC cells. **(A)** DKK4 knockdown (KD) activates JNK signaling, as detected by Western blot analysis. **(B)** DKK4 KD increases CRC cell sensitivity to 5-FU via JNK pathway activation. HCT116 cells were treated with 5 μM 5-FU and/or 0.5 μM JNK-IN-8 (JNK inhibitor, JNKi) for 4 days. Results are presented as the mean ± SD (n = 3), with p-values determined by a two-tailed t-test. **: *p* < 0.01, *** *p* < 0.001. **(C)** DKK4 KD promotes 5-FU induced apoptosis via JNK signaling activation. HCT116 cells were treated with 5 μM 5-FU and/or 0.5 μM JNK-IN-8 for 4 days.
